# The Anatomical Bill

**Published:** 1886-11-20

**Authors:** 


### THE ANATOMICAL BILL.

It has been a matter of consideration by previous Assemblies of our State to devise ways and means of supplying the material for anatomical study; and again this important matter has been brought to the attention of the General Assembly of the State of Georgia. A bill has been presented by the Hon. C. M. Candler, of DeKalb, entitled "An Act for the protection of Cemeteries and Burying Places in this State, and to prevent and punish the unauthorized uses of, and traffic in, dead human bodies, and for the promotion of Medical Science by the legal distribution and use of unclaimed dead human bodies for scientific purposes, through a Board created for that purpose, and for other purposes." It is stipulated that dead human bodies, required to be buried at public expense, shall be delivered to a Board of Distribution, to be used only within this State, solely for the advancement of Medical Science; provided, that no person intercsted in the deceased shall claim the body or bodies for burial, and, further, that the deceased was not a traveler, who has died suddenly, and that in all cases such body shall be held at least twenty-four hours after death before delivery to said board. An embarrasing condition is attached to this bill, that schools or colleges receiving such unclaimed dead bodies shall cause them to be embalmed and preserved intact for a period of ninety days, and shall deliver them to any person entitled to said body who may claim such body within the prescribed time. If, at the expiration of said period of ninety days, said bodies have not been claimed for burial they shall then be used by the medical schools and colleges for the purposes specified in the act; but the remnants shall be decently interred, and where this is not indicated, leaving this matter optionary. The Board may employ carriers for said bodies, who shall carefully deposit them, free from public observation, within suitable cases. The colleges are requircd to give a bond of $2,500 that the bodies received shall be used for the promotion of Medical Science within this State—which should serve as a sufficient guarantee. The traffic in dead human bodies beyond the State is made a felony, and punishable by imprisonment and hard labor, not less than three nor more than fiftcen years. Any one who removes a body after burial, or who receives such body, without the consent of the friends of said deceased, is guilty of felony, and punishable as above for grave-snatching. Thus, all the requirements for a faithful exeeution of this Act are plainly set forth, and it only remains to be seen whether it will become a law. There are points in this Act which are, in our judgment, objectionable, and we fear, if enacted, will defeat rather than accomplish the object desired.

